# Editorial: Emergent Public Health Issues in the US–Mexico Border Region

**DOI:** 10.3389/fpubh.2016.00093

**Published:** 2016-05-17

**Authors:** Cecilia Ballesteros Rosales, Scott Carvajal, Jill Eileen Guernsey de Zapien

**Affiliations:** ^1^Mel and Enid Zuckerman College of Public Health, University of Arizona, Tucson, AZ, USA

**Keywords:** border health, binational health, binational collaboration, US–Mexico border research

**The Editorial on the Research Topic**

**Emergent public health issues in the US–Mexico border region**

The public health literature in recent years has focused extensively on documenting and quantifying health disparities in the US. Race, ethnicity, and poverty are frequently considered the greatest predictors of inequality in health, and these factors are often inextricably tied to one another. Many studies have examined health disparities among those who live in relatively small geographical areas, such as inner cities, that are inhabited by minorities and/or the poor (1). However, in the case of the US–Mexico border region, a careful examination of data on health indicators in the 10 Border States, 4 in the US and the 6 along the northern border of Mexico, reveals both great disparities and strong similarities.

## Academic Response

Academic stakeholders in the US–Mexico region are committed to identifying gaps and/or responding to community appeals for solutions to both multifaceted and less complex questions. An example is the case of six universities in the US and Mexico that set out to stimulate collaborative, interdisciplinary scholarship, addressing border health issues relevant to public health in the region. The Puentes Consortium is represented by the Mexican institutions of Monterrey Tec, the University of Monterrey, and the University of the Americas in Puebla. The University of Arizona and Rice University; and as of 2014, University of California San Diego represents the US. The outcome of these efforts as well as a broader call to researchers for papers focused on border health is this special topic focused edition of 13 published articles in the journal Frontiers in Public Health.

The set of academic papers described in this supplement are significant examples of transborder partnerships addressing challenges characteristic of this region. They describe binational endeavors potentially translatable to promote programs and policies that improve population health. Moreover, they address and align with relevant and priority issues published in the US–Mexico Border Health Commission’s Healthy Border 2010/2020 Agenda (2). This work addresses broad themes in public health that span issues, such as chronic and infectious diseases, environmental health threats, cooperation of health providers and policymakers across borders, and structural and psychosocial factors, which relate to the health and well-being of marginalized populations in this region. We expect these articles will stimulate discussion and intensify the need for strategic action that can contribute to promoting health and well-being in the coming years in this border region. Further, the challenges, lessons learned, and opportunities from this work may be informative to others across the globe experiencing parallel dynamics, such as (1) the large-scale migration of marginalized populations resulting from economic and geopolitical instabilities in their homelands and (2) where nations near in physical proximity or with historical migratory corridors have vast disparities in resources, structures, and opportunities.

## Special Edition on Border Health

Three papers addressed challenges associated with the *monitoring and control of infectious diseases* as well as emerging diseases in the US–Mexico border region. Oren et al. investigated new tools to improve the diagnosis of latent TB. TB rates are elevated in the border region. Latent TB is of particular challenge as without symptoms or knowledge of infection, persons are unlikely to complete the rigorous medical regimen required to eliminate it. Yet, those affected (with some global prevalence estimates above 30%) are at risk for symptomatic TB and are infectious to others. Dengue has been a growing epidemic in Mexico and within the last 2 years there have been confirmed transmissions within Arizona. As mosquitoes that transmit dengue are not limited by physical and political boundaries, the paper by Arellano et al. illustrates the challenge where the interdependence of the knowledge and perceptions within border communities and public health agencies are particularly critical to the monitoring and prevention of potential outbreaks. The article by Valle et al. addressed a long-known border health challenge, HIV/AIDS. This work validated a measure of HIV stigma among men who have sex with men, particularly, disproportionally at risk for HIV/AIDS along the border region. Identification of such stigma in these men is critical for tailoring educational interventions aiming to reduce their risk behaviors and to optimize the likelihood that they will receive adequate medical services within one or both bordering nations they may travel between.

Five works focused on *health threats within marginalized and underserved populations*. As previously described, there was a study on the assessment of perceived HIV-associated stigma and its implications for HIV/AIDS prevention (Valle et al.). Influenced by the degree of national disasters such as Katrina and disproportionately affecting those without means to prepare or evacuate and with special medical needs, Meyer et al. surveyed a border coastal community. They found approximately 20% of the respondents have substantive medical special needs, and among them there is a diverse range of barriers that should be considered in disaster preparedness planning.

Multiple works also highlighted the vulnerability of migratory populations. Crocker elicited and synthesized testimonies of the first-generation (from Mexico) immigrants who are living in Arizona for an average of 15 years. Using a life-history approach, she identified extreme lifelong poverty, family separation, dangerous crossing experiences, and detention conditions as some of the common and major threats to the well-being of these immigrants. The paper by Valdez et al. also identified many parallel stressors and threats to mental health in Central American and Mexican migratory families released from short-term detention facilities in Southern Arizona. They also present a series of recommendations to reduce traumas exacerbated by current detention conditions and practices for immigrant men, women, and children.

The grounded-theory guided investigation of Sabo and Lee, who explored experiences and encounters with officials (border patrol, police, or military) of farmworkers, living and working on both sides of Arizona-Sonora. They found, regardless of legal/permanent status and migration status, these workers infrequently reporting authorities’ immigration enforcement-related abuses while frequently experiencing intense stress from interactions with these officials. Reasons for non-reporting include not knowing any mechanism to report violations to accountable officials, beliefs that individual violators would not be held accountable, risk for retaliation to themselves, friends, or family members, and due to the normalization or acceptance of these daily conditions. Finally, Stoesslé et al. investigated health concerns within a migratory sample in shelters in Northern Mexico of predominantly Central American undocumented immigrants. They identified persons needing basic health-care services and at high-risk for communicable diseases – including TB as well as experiencing symptoms of chronic disease. Further, fear with interactions with governmental services or agencies, low health literacy, and the effects of trauma from their homeland or journey were some of the major barriers to addressing those health needs.

Three papers addressed *barriers to improving health-care services in the border region or presented new public health models*. Matthews et al. presented the California Border Health Collaborative. This is a model to systematically promote coordination among hundreds of health-related organizations active in the California–Baja California region. These entities reflect various levels of government (federal, state, local) as well as higher education institutions, local non-profit organizations, and advocacy organizations. One key outcome of this collaboration model is to ensure policy makers are well informed of the implications of their policy decisions by stakeholders closest to those affected.

The following address more narrow but significant challenges to health-care delivery and coordination. Aristizabal et al. provide an innovative model to increase the capacity for a Mexican border hospital to improve cancer outcomes in children. Through partnering with two US-based border hospitals, a first of its kind (in the Tijuana area) pediatric oncology unit was launched. The disparity in 5-year survival from acute leukemia prior to this initiative (10% on the Mexican border side and 88% in the US border side) has been dramatically improved in the 6 years since implementation – with the rates 4–5 times better for Baja California children than prior. This successful model shows the high potential of cooperation of health services within Border States, when that effort is guided by evidence-based practice, cultural responsiveness, developing trust, and respectful interchange. Chronic disease risks also remain disproportionally high for Hispanics living in the border region relative to Hispanics living in other regions of the US and Mexico. De Heer et al. report on a community health worker-delivered intervention in a large US city within the border region. Participants in this intervention were able to use community resources to promote healthy lifestyle and showed improvements in physical activity, diet, and clinical indicators (e.g., weight, blood pressure). Using the relatively lower costs of community health workers (relative to other health-care professionals) and by leveraging existing community resources, this intervention is likely scalable to reduce CVD risk within Mexican-origin populations in both the nations.

In concluding the special topic issue, two papers address *the role of broader social conditions and environments* on health issues within border populations. Valdez and Langellier examined mental health problems and services within Arizona and focused on differences in Whites and Hispanics. They found Hispanics reported lower mental health diagnosis than Whites, though in both groups, lower SES was associated with greater likelihood of distress. Their findings suggest more undiagnosed cases of mental health conditions in Hispanics and that more culturally and linguistically appropriate strategies to provide mental health diagnosis and services is needed in Arizona. Salinas and Sexton investigated food environment – which could contribute to obesity, diabetes, and CVD risk with community-level ethnic density and poverty. They not only identified some urban border environments appearing as health protective relative to non-border urban minority communities in Texas but also concluded any positive effects may be attenuated by other factors that need to be further explored (e.g., they may be available but costly to low-income residents).

The papers summarized here are exemplars of the high level of collaboration essential to conducting and disseminating public health research that translates into action to improve health and well-being in border communities.

## Author Contributions

The lead author is CR. CR outlined and drafted the editorial. SC and JZ contributed by reviewing and revising the manuscript.

## Conflict of Interest Statement

The authors declare that the research was conducted in the absence of any commercial or financial relationships that could be construed as a potential conflict of interest.
